# A Case Report on a Giant Hepatic Inflammatory Adenoma in a Young Female That Presented as Spontaneous Intrahepatic Hematoma

**DOI:** 10.7759/cureus.42055

**Published:** 2023-07-18

**Authors:** Andreas Kyvetos, Panagiota Voukelatou, Ioannis Vrettos, Spyridon Pantzios, Ioannis Elefsiniotis

**Affiliations:** 1 Second Department of Internal Medicine, General and Oncology Hospital of Kifissia “Agioi Anargyroi”, Athens, GRC; 2 Academic Department of Internal Medicine-Hepatogastroenterology Unit, General and Oncology Hospital of Kifissia “Agioi Anargyroi”, Athens, GRC

**Keywords:** mri, ct scan, benign liver tumors, spontaneous intrahepatic hematoma, hepatic inflammatory adenoma

## Abstract

Among the extensive variety of disorders that can cause acute abdominal pain are hepatocellular adenomas (HCAs), pathological entities that otherwise are asymptomatic. Here, we describe a 33-year-old female who presented in the emergency department with acute abdominal pain and a history of liver focal nodular hyperplasia (FNH) diagnosed 10 years ago. An abdominal magnetic resonance imaging (MRI) revealed that the cause of the pain was an intrahepatic hematoma. The mass was surgically removed, and the biopsy revealed inflammatory adenomas, a subtype of HCA. Hepatic adenoma diagnosis remains challenging by clinical and imaging techniques, and usually, a biopsy is the main diagnostic tool. HCA should be differentiated from hepatocellular carcinoma (HCC), FNH, hepatic angioleiomyoma, and hepatic hemangioma. In our case, HCA was misdiagnosed in the past as FNH. HCA rarely may present as acute right abdomen pain, and a potential catastrophic hemorrhage or rupture must be excluded.

## Introduction

With the growing use of various imaging modalities in the diagnosis of abdominal and other symptoms, liver masses represent a relatively frequent finding [1]. Liver masses can be classified into three categories: benign asymptomatic lesions, benign symptomatic lesions, and malignant neoplasms [2]. If no history of chronic liver disease or known neoplasia is present, then the diagnosis of a solitary liver lesion is most likely hemangioma, focal nodular hyperplasia (FNH), and hepatocellular adenoma (HCA) [2]. FNH is the second most frequent benign lesion after hemangiomas with a prevalence of 2.5%. The presence of a central fibrotic scar is the main characteristic finding on computed tomography (CT) scans. The clinical presentation is uneventful with no severe complications, so no treatment is recommended [2]. HCAs are very uncommon with a reported prevalence of 0.001. They are diagnosed usually in women aged 35-40 years old (female/male ratio: 10:1) [3] and are usually a solitary lesion [2]. The diagnosis of HCA is challenging as it is usually misdiagnosed through clinical and imaging examinations [4]. The differential diagnosis between HCA and FNH is challenging even with the most sensitive imaging techniques, so a biopsy is usually the only solution [2].

We report a case report of a 33-year-old female who presented at the emergency department with pain in the right upper quadrant of the abdomen. A mass measuring 19 × 14 cm was found on the right liver lobe.

This article was previously presented as a meeting abstract at the 21st European Congress of Internal Medicine and the 12th International Congress of Internal Medicine, Athens, Greece, on March 15-18, 2023.

## Case presentation

We present a case of a 33-year-old Caucasian female patient with a history of anxiety disorder treated with sertraline 100 mg once daily and liver focal nodular hyperplasia (FNH) diagnosed 10 years ago without follow-up. Additionally, she was diagnosed with polycystic ovary syndrome and received a combined oral contraceptive pill for a small period of time, which she discontinued a few years ago.

She presented at the emergency department with 24-hour pain in the right upper quadrant of the abdomen. The pain was getting worse with breathing movements. Initial laboratory analysis showed elevated white blood count (WBC) and abnormal levels of liver enzymes (Table 1).

**Table 1:** Initial laboratory analyses WBC = white blood cell count, NEU = neutrophils, Hb = hemoglobin, PLT = platelet count, INR = international normalized ratio, CRP = C-reactive protein, AST = aspartate aminotransferase, ALT = alanine transaminase, GGT = gamma-glutamyl transferase, ALP = alkaline phosphatase, LDH = lactate dehydrogenase

Parameter	Value	Normal range
WBC	14.4 × 10^3^/μL	4-11 × 10^3^/μL
NEU	10.69 × 10^3^/μL	2.5-7.5 × 10^3^/μL
Hb	10.4 g/dL	12-16 g/dL
PLT	654 K/μL	150-450 K/μL
INR	0.91	0.85-1.2
Ferritin	1,264 ng/mL	11-204 ng/mL
CRP	11.22 mg/dL	<0.5 mg/dL
Total bilirubin	1.1 mg/dL	0.2-1.0 mg/dL
Direct bilirubin	0.4 mg/dL	<0.3 mg/dL
AST	393 U/L	9-36 U/L
ALT	173 U/L	10-28 U/L
GGT	142 U/L	9-30 U/L
ALP	69 U/L	42-141 U/L
LDH	711 U/L	230-460 U/L

**Figure 1:**
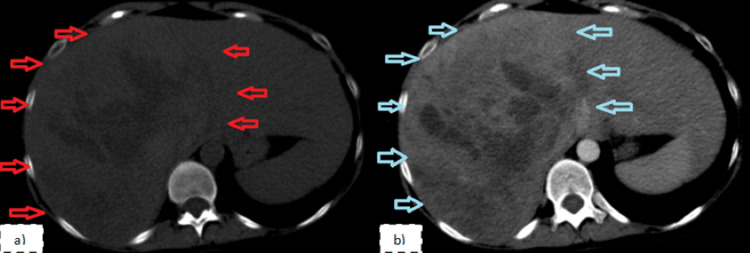
Abdominal CT scan (a) Unenhanced scan. (b) Arterial phase of contrast-enhanced scan. It revealed a giant heterogeneous hepatic tumor with a clear boundary measuring 19 × 14 cm, which completely replaced the right hepatic lobe (red and blue arrows). The intravenous contrast medium showed patchy enhancement. CT = computed tomography

An abdominal CT scan with contrast was performed that revealed a giant heterogeneous hepatic tumor with clear boundary measuring 19 × 14 cm, which completely replaced the right hepatic lobe. The contrast showed patchy enhancement (Figure 1).

**Figure 2:**
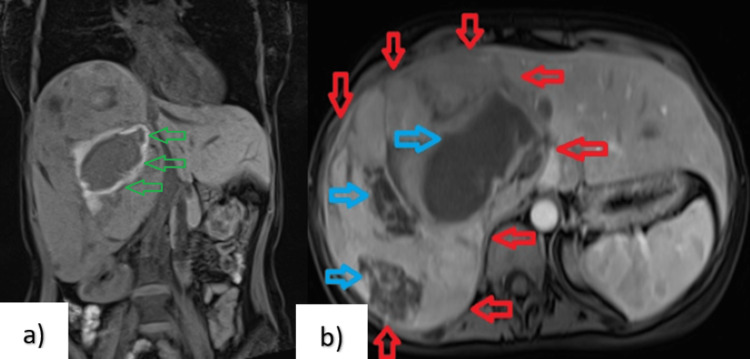
Abdominal MRI scan (a) MRI coronal view pre-contrast T1-weighted imaging compatible with hemorrhage (green arrows). (b) MRI view contrast T1-weighted imaging revealed a large (red arrows), lobulated, heterogeneous (blue arrows) right hepatic lobe lesion. MRI = magnetic resonance imaging

The patient was prescribe ciprofloxacin 400 mg twice daily and metronidazole 500 mg three times daily and had remarkable improvement in her symptoms and her blood examination with normalization of WBC and C-reactive protein (CRP) values within a few days, and the level of hemoglobin (Hb) remains the same. To further diagnose the patient’s condition, magnetic resonance imaging (MRI) was also performed that revealed a giant heterogeneous lesion on the right hepatic lobe with echo sequences of intrahepatic hemorrhagic elements (Figure 2).

The patient had CT scan and MRI images from 10 years ago that revealed that the mass was getting bigger. According to the diagnosis 10 years ago, the mass was a liver focal nodular hyperplasia. Based on our imaging manifestations, the tumor was initially considered to be an HCA.

**Figure 3:**
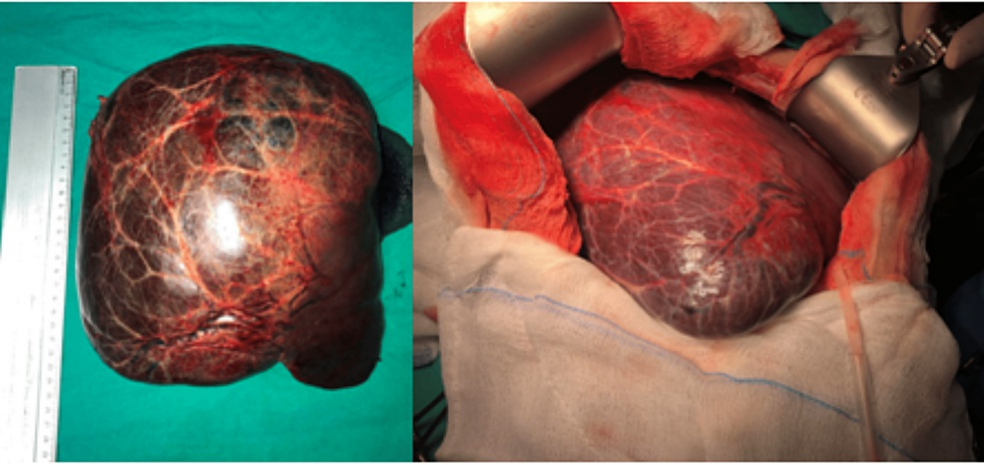
Perioperative images

At this time, the admission to the surgery room was decided to completely remove the mass, and a right hepatic lobectomy was performed. Perioperative images showed a grayish-yellow tumor with a complete fibrous capsule and varying degrees of hemorrhage and necrosis (Figure 3). Pathology examination revealed a concentration of inflammation, thick arteries, and sinusoidal dilatation, the main histological features of inflammatory adenomas [5]. The patient recovered without complications. A six-month follow-up was performed without any symptoms or clinical signs.

## Discussion

HCA can remain asymptomatic for many years, and the diagnosis of this benign tumor is mainly made by imaging [4]. In our case, it presented as acute abdominal pain. The pain probably is associated with the torsion of the vascular pedicle, intra-abdominal hemorrhage, or intrahepatic hematoma as in our case [6]. The clinical presentation of HCA is not uneventful as around 25% of patients report mild abdominal pain. The most frequent complication of HCA is necrosis and bleeding especially in a patient with a history of prolonged oral contraceptive pills [2]. Hemorrhage is reported with an overall frequency of 27.2% of HCA lesions [7].

The major risk factors for HCA other than oral contraceptive pills are metabolic syndrome, obesity, alcohol intake, and the use of anabolic steroids, which in our case were absent [5]. Estrogen exposure is a risk factor, as there exists a strong association between the use of high-dose first-generation oral contraceptives and the development of HCA [8]. Nevertheless, in some cases, a reduction in size is observed following optimal metabolic control [3]. The dimensions of HCA increase likely because of the use of oral contraceptive pills.

Four subtypes of HCA exist: (1) HCA inactivated for HNF-1a (30%-40%), (2) inflammatory adenomas (40%-50%), (3) β-catenin-activated HCA (10%-20%), and (4) unclassified HCA (5%-10%). The mass in our case had a concentration of inflammation, thick arteries, and sinusoidal dilatation, the histological features of inflammatory adenomas [5]. Molecular subtyping, especially activating mutations in β-catenin, is correlated with the risk of malignant transformation into hepatocellular carcinoma (HCC) [3,9]. The addition of β-catenin immunohistochemistry was performed, and it was negative for our case.

HCA should be differentiated from hepatocellular carcinoma (HCC), focal nodular hyperplasia (FNH), hepatic angioleiomyoma, and hepatic hemangioma [10-12]. The importance of the differential diagnosis between FNH and HCA is the difference in treatment management as the FNH required no treatment [2]. The main pathologic feature of HCA is the presence of fat or telangiectatic components. In CT scans, HCAs are usually hypoattenuating but may appear hyperattenuating based on the extent of fatty infiltration of the liver parenchyma. In contrast CT scans, HCAs are usually heterogeneous [13]. In our case, the tumor was misdiagnosed in the past as FNH. In contrast, the CT scan of FNH is characterized by uniform enhancement, except in scar tissue [14]. Additional FNH is a tumor without a capsule or hemorrhagic necrosis in contrast to our case [4]. So, the mass had a capsule and intrahepatic hematoma and was getting bigger with oral contraceptives, characteristics of an HCA. As demonstrated, CT scan and MRI images are essential for the diagnosis.

The management plan of HCA is different between males and females. Recent guidelines suggest surgical resection in all sizes of tumors in males [3]. In females, surgical resection is suggested if the tumor size is over 5 cm [4]. In our case, except for the intrahepatic hematoma, the patient had surgical indications because of the giant tumor size. For smaller tumor sizes of less than 5 cm in females, conservative management with cessation of contraceptive pills, anabolic steroids, weight loss, and repeated periodic imaging is suggested [4].

## Conclusions

We report a case of a giant HCA in a young female that presented as acute abdominal pain due to spontaneous intrahepatic hematoma. HCA is a rare benign tumor that may present as acute right abdominal pain, and a potential catastrophic hemorrhage or rupture must be excluded. Hepatic adenoma diagnosis remains challenging by clinical and imaging techniques, and usually, a biopsy is the main diagnostic tool. In our case, CT scan and MRI images were essential for the diagnosis of HCA. Special attention must be given to patients with a history of a benign liver mass and oral contraceptive pill use.
